# Supplementary material for: Recording vaccine doses administered: A global analysis of tally sheet design for infant and child immunizations
Source: PLOS Glob Public Health. 2025 Oct 6;5(10):e0004973. doi: 10.1371/journal.pgph.0004973 (PMC12500165; doi:10.1371/journal.pgph.0004973)
Supplement: Attachment — Submitted filename: Review comments_Recording vaccine doses administered.DOC [file pgph.0004973.s002.DOC]

Manuscript: PGPH-D-25-01834

Title: Recording vaccine doses administered: A global analysis of tally sheet design for infant and child immunizations

**Review comments**

Firstly, thank you very much for providing the opportunity to review this manuscript. Second, I will list out all the important comments to be incorporated within the manuscript for a better research output.

**ABSTRACT**

1. Page 2: line 21-37: Divide the abstract into components such as Introduction, Objective, Methodology, Result, Conclusion and recommendations to see the abstract of the article clearly.
2. Page 2: line 38-39. Add the keywords used the article not more than 8 words.

**Introduction**

3. Page 4: line 64-73: Important points were missed about the significance of this study. What could be the significance of this research for healthcare workers, program managers, programmers, policy makers etc. at national and subnational levels? What would also the significance of using standard tally sheets across different countries? These points should be aggressed.

**Materials and methods**

4. Page 4: line 78-79: You said that a total of 208 reporting entities submitted eJRF data. Does it mean one reporting entitled from each country or multiple tally sheets submitted in a single country. It needs further clarifications.
5. Page 4: line 83-84: What about the catch up vaccination tally sheet since it has difference in age group, antigen type that depends on vaccinated child etc…?
6. Page 5: line 89-90: Some countries tally sheet may embedded with monthly reports. Therefore, how you managed the selection biases?
7. Page 5: line 91-101: What are your exclusion criteria’s? The exclusion criteria’s should be set at methodology of your article before exclusion of data.
8. Page 6: line 106-107: Of the retained tally sheets 49 were for WHO member states and 2 were from other reporting entities. Countries with out WHO member states are not well represented. In addition, WHO member states would have more technical support from WHO. Therefore, how do you see those biases in data selection process?
9. Page 5: line 103-104 footnote and page 6: line 106-107 are the same sentences. Therefore, please remove one of them.
10. Page 6: line 108-116: Please include this paragraph at the limitation of the study component of the article.
11. Page 8-9: line 135-140: Each figure should be labeled.
12. Page 8-9: line 135-140: With WHO recommendation guidelines, many countries are not providing BCG vaccines more than 1 years of child age. However, on the tally sheets show the provision of BCG vaccine older than 1-year children. Make sure that those tally sheets are reliable.
13. Page 10: line 164-165: This paragraph is repetitive. It was written from line 153-160. Therefore, remove repetitive results from the result section.
14. Page 10: line 172-173: All table titles should have a time period, sample size, clear heading, informative and allowing the article readers to quickly understand the table content. Please update the title of all tables.

**Discussion**

15. Page 18: line 299-301: The authors has explained about the catch up and big catch up vaccinations here without explaining at the introduction of your article. Please update it at the introduction regarding to the catch up and big catch up vaccinations.
16. Page 18-20: line 276-349: Separate the discussion from conclusion and recommendations.
17. Page 18-20: line 276-349: Add the strength and limitation of the study.
18. Page 21: line 367-368: Add the acknowledgement section within the manuscript. At least the Ministry of Health of data rouses should be acknowledged.

**Overall comment**

This study is well-conducted, and the researchers are commended for their work thorough evaluating Recording vaccine doses administered: A global analysis of tally sheet design for infant and child immunizations. The study is effectively structured, with a clear identification of the impact of tally sheet design for infant and child immunizations in different countries. However, to enhance its credibility and ensure clear communication of the findings, the researchers should address the minor comments and suggestions provided above. Once these revisions are made, the study will be ready for publication and will provide valuable insights for infant and child immunizations.
