# Supplementary material for: Recording vaccine doses administered: A global analysis of tally sheet design for infant and child immunizations
Source: PLOS Glob Public Health. 2025 Oct 6;5(10):e0004973. doi: 10.1371/journal.pgph.0004973 (PMC12500165; doi:10.1371/journal.pgph.0004973)
Supplement: Attachment — Submitted filename: Response to Reviewers.docx [file pgph.0004973.s004.docx]

29 August 2025

Dear Editors and Reviewers,

Thank you for the constructive feedback on our manuscript, PGPH-D-25-01834 titled Recording vaccine doses administered: A global analysis of tally sheet design for infant and child immunizations.

We have carefully revised the manuscript to address all comments and believe the changes have strengthened it. Below, we provide a point-by-point response outlining how each remark has been addressed in the revision. Lines number references in our responses refer to the track change version of the manuscript. We have also noted three changes to references.

*Point-by-point responses to reviewer feedback:*

**Reviewer #1**

**Comment:** *Page 2: line 21-37: Divide the abstract into components such as Introduction, Objective, Methodology, Result, Conclusion and recommendations to see the abstract of the article clearly.*

**Response:** The abstract has been revised accordingly.

**Comment:** *Page 2: line 38-39. Add the keywords used the article not more than 8 words.*

**Response:** We have added key words.

**Comment:** *Page 4: line 64-73: Important points were missed about the significance of this study. What could be the significance of this research for healthcare workers, program managers, programmers, policy makers etc. at national and subnational levels? What would also the significance of using standard tally sheets across different countries? These points should be addressed.*

**Response:** Thank you for raising. We have added points on the significance of this study to the first paragraph in the discussion section.

There are some important elements of tally sheets that the paper mentions, i.e. site and session characteristics (date, place, delivery modality), national vaccine schedule, and programmatic priorities; however, due to different vaccine schedules and recommendations across countries, further standardization across countries is not recommended. This is part of the significance and contribution of this paper in discussing and pointing to guidance documents where vaccine or programme specific information are found.

**Comment:** *Page 4: line 78-79: You said that a total of 208 reporting entities submitted eJRF data. Does it mean one reporting entitled from each country or multiple tally sheets submitted in a single country. It needs further clarifications.*

**Response:** There were a total of 214 member states and reporting entities, 208 reported to the eJRF among which a subset of 71 uploaded a file to the question on tally sheets and 51 submissions were retained (Figure 1). Lines 107-108, in tracked changes document revision, have been revised for more clarity that the 51 submissions are separate submissions with no duplicates or geographic overlap.

**Comment:** *Page 4: line 83-84: What about the catch up vaccination tally sheet since it has difference in age group, antigen type that depends on vaccinated child etc…?*

**Response:** Thank you, we added clarifying language to line 85.

**Comment:** *Page 5: line 89-90: Some countries tally sheet may embedded with monthly reports. Therefore, how you managed the selection biases?*

**Response:** Selection was managed through the inclusion and exclusion criteria added in this revision (lines 89-95) in response to the related comment below.

**Comment:** *Page 5: line 91-101: What are your exclusion criteria’s? The exclusion criteria’s should be set at methodology of your article before exclusion of data.*

**Response:** Thank you for this observation. We have included more explicit inclusion and exclusion criteria in lines 89-95.

**Comment:** *Page 6: line 106-107: Of the retained tally sheets 49 were for WHO member states and 2 were from other reporting entities. Countries with out WHO member states are not well represented. In addition, WHO member states would have more technical support from WHO. Therefore, how do you see those biases in data selection process?*

**Response:** This is the first global or cross-country analysis of tally sheets and while a larger sample would provide more representative results across WHO member status and World Bank income classifications, we see this as an important evidence summary filling of available submissions and discussion of related guidance and approaches. It is the start of filling a major gap in implementation literature on immunization recording tool design.

**Comment:** *Page 5: line 103-104 footnote and page 6: line 106-107 are the same sentences. Therefore, please remove one of them.*

**Response:** Thank you, the in-text sentence has been removed and the footnote retained.

**Comment:** *Page 6: line 108-116: Please include this paragraph at the limitation of the study component of the article.*

**Response:** We agree, and the limitations paragraph has been moved to the Discussion section (last paragraph of the Discussion and before the Conclusion section).

**Comment:** *Page 8-9: line 135-140: Each figure should be labeled.*

**Response:** Edits have been made to Figure 2 a–c labels and can be further adjusted. These three figures (illustrative examples of tally sheet designs) are intended to be shown as a group to show design differences. Each is labelled a, b, c with the prominent design feature mentioned in the title.

**Comment:** *Page 8-9: line 135-140: With WHO recommendation guidelines, many countries are not providing BCG vaccines more than 1 years of child age. However, on the tally sheets show the provision of BCG vaccine older than 1-year children. Make sure that those tally sheets are reliable.*

**Response:** With reference to [WHO Recommendations for Interrupted or Delayed Routine Immunization](https://www.who.int/publications/m/item/table-3-recommendations-for-interrupted-or-delayed-routine-immunization-summary-of-who-position-papers), updated December 2024, it does not specify an upper age threshold for BCG and indicates that BCG is recommended for unvaccinated older children, adolescents and adults from settings with high incidence of TB and/or leprosy burden (see pages 1 and 4). BCG vaccination of unvaccinated older aged groups is recommended in the WHO position paper 2018 (page 94) which informs the current global recommendations. Please see: World Health Organization. BCG vaccines: WHO position paper – February 2018. Wkly Epidemiol Rec. 2018;93(8):73-96. Available from: <https://www.who.int/publications/i/item/who-wer9308-73-96>

**Comment:** *Page 10: line 164-165: This paragraph is repetitive. It was written from line 153-160. Therefore, remove repetitive results from the result section.*

**Response:** The text is revised to remove the repetition.

**Comment:** *Page 10: line 172-173: All table titles should have a time period, sample size, clear heading, informative and allowing the article readers to quickly understand the table content. Please update the title of all tables.*

**Response:** Thank you, we have added the requested information in the titles for each table.

**Comment:** *Page 18: line 299-301: The authors has explained about the catch up and big catch up vaccinations here without explaining at the introduction of your article. Please update it at the introduction regarding to the catch up and big catch up vaccinations.*

**Response:** The Big Catch-up is mentioned in the Introduction section (lines 62-63) and cites two sources for further information.

**Comment:** *Page 18-20: line 276-349: Separate the discussion from conclusion and recommendations.*

**Response:** A Conclusion sub-heading has been added (line 389).

**Comment:** *Page 18-20: line 276-349: Add the strength and limitation of the study.*

**Response:** The strengths have been added (lines 368-377), followed by the paragraph on limitations.

**Comment:** *Page 21: line 367-368: Add the acknowledgement section within the manuscript. At least the Ministry of Health of data rouses should be acknowledged.*

**Response:** Thank you for this observation. We have added this section and specifically thanked the Ministries of Health who submitted an example of their tally sheet included in this analysis (lines 395-398) as well as a colleague who provided feedback on the manuscript.

**Overall comment:** *This study is well-conducted, and the researchers are commended for their work thorough evaluating Recording vaccine doses administered: A global analysis of tally sheet design for infant and child immunizations. The study is effectively structured, with a clear identification of the impact of tally sheet design for infant and child immunizations in different countries. However, to enhance its credibility and ensure clear communication of the findings, the researchers should address the minor comments and suggestions provided above. Once these revisions are made, the study will be ready for publication and will provide valuable insights for infant and child immunizations.*

**Response:** We thank the reviewer for taking the time to review and provide very useful feedback on our paper.

**Reviewer #2**

**Comment:** *The presented research article "Recording vaccine doses administered: A global analysis of tally sheet design for infant and child immunizations" contributes original research closely related to current literature. It addresses one of contemporary main challenges in immunisation, particularly in low-income countries, i.e., immunisation data quality. Accuracy and completeness of these data are essential for vaccination coverage monitoring, identifying missed opportunities, following up progress, and guiding correction measures for vaccination teams.

The study utilises open international data and qualitative and quantitative analysis. Used data processing algorithm with reproducibility has been made available by researchers, and additional tables are given. Results obtained are valuable and useful, specifically because data sources come from around the globe. However, since completeness in data varied significantly from one country to another, making robust generalizable inferences is not possible. Despite this limitation, the observations remain valuable for future work as well as in policy debates.
The article has not been published previously and it has been posted only at a preprint server, which is in line with PLOS's policy.*

**Response:** We thank the reviewer for this valuable feedback.

**Comment:** *Line 159: Change C to “See.”*

**Response:** Language revised to refer to Figure 2c.

**Comment:** *Line 233: Change “who’s” for "whose"*

**Response:** The line is in quoted text followed by the Latin grammatical reference [sic] indicating that the quoted passed contains an error. We suggest maintaining as is unless there is a preferred editorial approach.

**Comment:** *Line 233: Define sic before first use.*

**Response:** We suggest to maintain as is unless there is a preferred editorial approach.

**Comment:** *Line 302 and 306: Change existing quotation ' ' marks to “” to maintain consistency.*

**Response:** Thank you, the lines are revised accordingly (lines 321 and 325).

**Comment:** *Line 342: keep the consistent hyphenation of “stock-outs” versus “stockouts.”*

**Response:** Thank you, the line is revised accordingly.

**Additional changes**

**Change:** Reference 12 (a duplicate to reference 15) has been replaced.

**Change:** Two additional sources have been added – new 30 and 31 – and subsequent references have been renumbered.
